# Tailored interventions to prevent functional decline after a Sentinel Fall presenting to the Emergency Department (iSeFallED) – protocol for a pragmatic mixed-methods implementation study

**DOI:** 10.1186/s12877-025-06943-0

**Published:** 2026-01-07

**Authors:** Tania Zieschang, Laura Himmelmann, Nina Marie Schmidt, Elisa-Marie Speckmann, Lea Feld, Jochen Klenk, Thea Laurentius, Kathrin Boerner, Rebecca Diekmann, Andreas Hein, Jessica Koschate-Storm, Lars Schwettmann, Milena von Kutzleben, Tim Stuckenschneider

**Affiliations:** 1 Geriatric Medicine, Department for Health Services Research, School of Medicine and Health Sciences, Carl von Ossietzky University Oldenburg, Ammerländer Heerstraße 114-118, Oldenburg, 26129 Germany; 2 Assistance Systems and Medical Device Technology, Department for Health Services Research, School of Medicine and Health Sciences, Carl von Ossietzky University, Oldenburg, Germany; 3 Department of Medical Physics and Acoustics Medical Physics, Cluster of Excellence Hearing4all Carl von Ossietzky University, Oldenburg, Germany; 4 Institute of Epidemiology and Medical Biometry, Ulm University, Ulm, Germany; 5 Prevention and Rehabilitation Research, Department for Health Services Research, School of Medicine and Health Sciences, Carl von Ossietzky University, Oldenburg, Germany; 6 Junior Research Group Nutrition and Physical Function in Older Adults, Department for Health Services Research, School of Medicine and Health Sciences, Carl von Ossietzky University, Oldenburg, Germany; 7 Division Health Economics, Department for Health Services Research, School of Medicine and Health Sciences, Carl von Ossietzky University, Oldenburg, Germany; 8 Mixed Methods-Lab in the Division for Prevention- and Rehabilitation Research, Department of Health Services Research, School of Medicine and Health Sciences, Carl von Ossietzky University of Oldenburg, Oldenburg, Germany

**Keywords:** Falls prevention, Older adults, Machine learning, Patient involvement, Secondary prevention, Perturbation, Activities of daily living, Cognitive impairment, Strength training, Focus groups, Hearables

## Abstract

**Background:**

Falls are a leading cause of emergency department (ED) presentations among older adults and frequently signal the onset of functional decline, reduced mobility, and recurrent falls. While evidence-based falls prevention strategies, particularly strength and balance training, can substantially reduce fall risk, secondary prevention is rarely initiated in ED settings. Building on insights from the observational SeFallED study, the iSeFallED study aims to implement an individualized secondary falls prevention program directly within the ED. The intervention integrates comprehensive geriatric assessment, tailored exercise options, wearable sensor-based monitoring, and perturbation-based treadmill training, combined with participatory research methods to ensure patient-centered refinement.

**Methods:**

iSeFallED is a pragmatic mixed-methods implementation trial enrolling adults aged 60 years and older who present to the ED of the Klinikum Oldenburg or the Evangelisches Krankenhaus Oldenburg following a fall and are discharged without hospital admission. A risk stratification algorithm developed from SeFallED data assigns participants to one of three intervention arms. Individuals classified as having mild risk for functional decline receive educational materials on physical activity and falls prevention. Participants identified as having at least moderate risk may choose between a home-based, tablet-guided strength and balance program or supervised group-based training delivered by local sports partners or at the university center. Optional treadmill perturbation-based balance training is available to all intervention groups. Assessments occur at baseline and at 6, and 12 months, capturing activities of daily living, functional performance, fall risk factors, quality of life, physical activity, and fall incidence. Continuous activity and mobility data are collected through wearable sensors, while focus groups with participants, caregivers, and stakeholders capture qualitative insights. A target sample size of 350 participants will enable comparison with the historical SeFallED sample, with change in activities of daily living serving as the primary outcome. Secondary outcomes include recurrent falls, mobility, and adherence to intervention pathways.

**Discussion:**

The iSeFallED study will provide evidence on the feasibility of initiating secondary falls prevention in the ED and will evaluate its efficacy relative to standard care using a historical control group. By identifying barriers and facilitators to implementation and incorporating machine learning based analysis of wearable sensor data, the study aims to refine secondary falls prevention strategies and offer a scalable model for integration into challenging clinical environments such as the ED.

**Trial registration:**

Prospectively registered on 5 March 2025 in the Deutsches Register für Klinische Studien, (DRKS00035322; Date of registration in DRKS: 2025–03 – 05).

**Supplementary Information:**

The online version contains supplementary material available at 10.1186/s12877-025-06943-0.

## Background

The total number of deaths and loss of disability-adjusted life years attributed to falls has shown a steady increase in Europe since 1990 [1]. According to the Global Burden of Disease study, falls accounted for nearly 17 million years of life lost in 2017 [2]. In ageing societies, the societal and economic impacts of falls are profound, with fall-related expenses comprising approximately 1% of healthcare costs in high-income countries [3]. Consequently, primary and secondary falls prevention have become critical global healthcare priorities, aiming to reduce fall incidence and mitigate long-term consequences [4].

In 2022, a global task force synthesized existing evidence on falls prevention and management, resulting in comprehensive guidelines with evidence-based and expert-driven recommendations [5]. These guidelines present an algorithm for risk stratification in older adults, identifying high-risk individuals, such as those presenting to emergency departments (EDs), as priority candidates for tailored interventions. Multifactorial risk assessments guide these interventions, enabling personalization to meet individual needs. Exercise programs, particularly those emphasizing balance and strength training, are the most strongly supported strategies, backed by high-quality evidence [5]. Innovative approaches, such as treadmill perturbation-based balance training, should also be considered for integration into clinical practice, given their demonstrated efficacy with fewer stimuli [6, 7].

Despite the robust evidence supporting traditional exercise-based interventions, their practical implementation in clinical settings remains poorly defined. The global guidelines underscore the importance of adapting strategies to local contexts and available resources, yet further research is required to develop and evaluate effective implementation concepts to address this gap [5].

Limited research has examined the implementation of falls prevention in the ED, a critical setting as it frequently serves as the initial – and sometimes only – point of contact for patients after a fall. Falls are a leading cause of ED presentations [8], yet the unique characteristics of the ED – marked by overcrowding, urgent priorities, and limited staff capacity – pose significant challenges to integrating prevention measures [9, 10]. A systematic review on multifactorial falls prevention in the ED suggests that reductions in fall incidence are unlikely to be achieved through referral-based programs alone, but effective approaches require more than a single patient encounter and should incorporate direct treatment or multiple interactions. Moreover, interventions need to be specifically adapted to the care needs of this population group [11]. However, the heterogeneity of patients presenting to the ED complicates the identification and delivery of individualized interventions [12].

A randomized controlled pilot trial conducted in the United States demonstrated that integrating a pharmacist and physiotherapist into ED care for targeted risk assessments reduced ED revisits within six months [13]. While promising, resource constraints limit the scalability of such interventions [14–16], emphasizing the need for alternative solutions. Notably, a key guideline recommendation – strength and balance training, recognized as the most effective intervention for falls prevention – was not implemented in this study. A potential strategy to enhance individualized care while maintaining feasibility within the ED setting is the incorporation of brief risk assessments into standard ED workflows, with high-risk patients referred to specialized falls clinics [17]. Conceptual workflows in previous research suggest that falls clinics could bridge the gap between ED care and secondary prevention, providing a more sustainable and targeted approach to managing falls risk [17].

When designing and implementing such care pathways, it is essential to align interventions with the specific needs of the target population to ensure adherence [18]. Patient-centered approaches, which address barriers and facilitators to participation, are critical for ensuring program compliance. Exploring the perspectives of patients, healthcare providers, and exercise professionals is vital to creating feasible, clinically applicable and tailored intervention programs [19, 20].

The iSeFallED study aims to address these challenges by implementing tailored and scalable secondary prevention into clinical practice. Furthermore, it will evaluate the feasibility and acceptability of a rapid risk assessment tool in the ED, followed by a comprehensive geriatric assessment in a specialized falls clinic and a six-month intervention period. It will also assess the efficacy with respect to maintaining independence in the activities of daily living post-fall, while exploring the effects of different exercise interventions on secondary outcomes such as quality of life and falls incidence. In addition, the iSeFallED study will investigate the feasibility of treadmill perturbation-based balance training in a clinically diverse and heterogenous population. By integrating sensor-derived data with machine learning methods for the detection of gait disturbances, and by examining individual barriers, preferences, and needs, iSeFallED aims to optimize the implementation process and evaluate the feasibility of initiating secondary falls prevention in the ED setting.

## Methods

### Study design

The iSeFallED study is a pragmatic implementation trial utilizing a mixed-methods approach to evaluate the feasibility, acceptability, and effectiveness of a secondary falls prevention intervention for older individuals following a fall with presentation to the ED without subsequent hospitalization. Previous studies have reported participation rates of approximately 50% in falls prevention programs at 12 months [21]. To enhance adherence, participants in the current study are allowed to choose their preferred intervention. Consequently, a randomized controlled trial (RCT) design was deemed infeasible. Instead, a quasi-experimental design will be used, comparing outcomes with a historical control group from the prior observational SeFallED study, conducted between 2021 and 2025 [22].

**Fig. 1:**
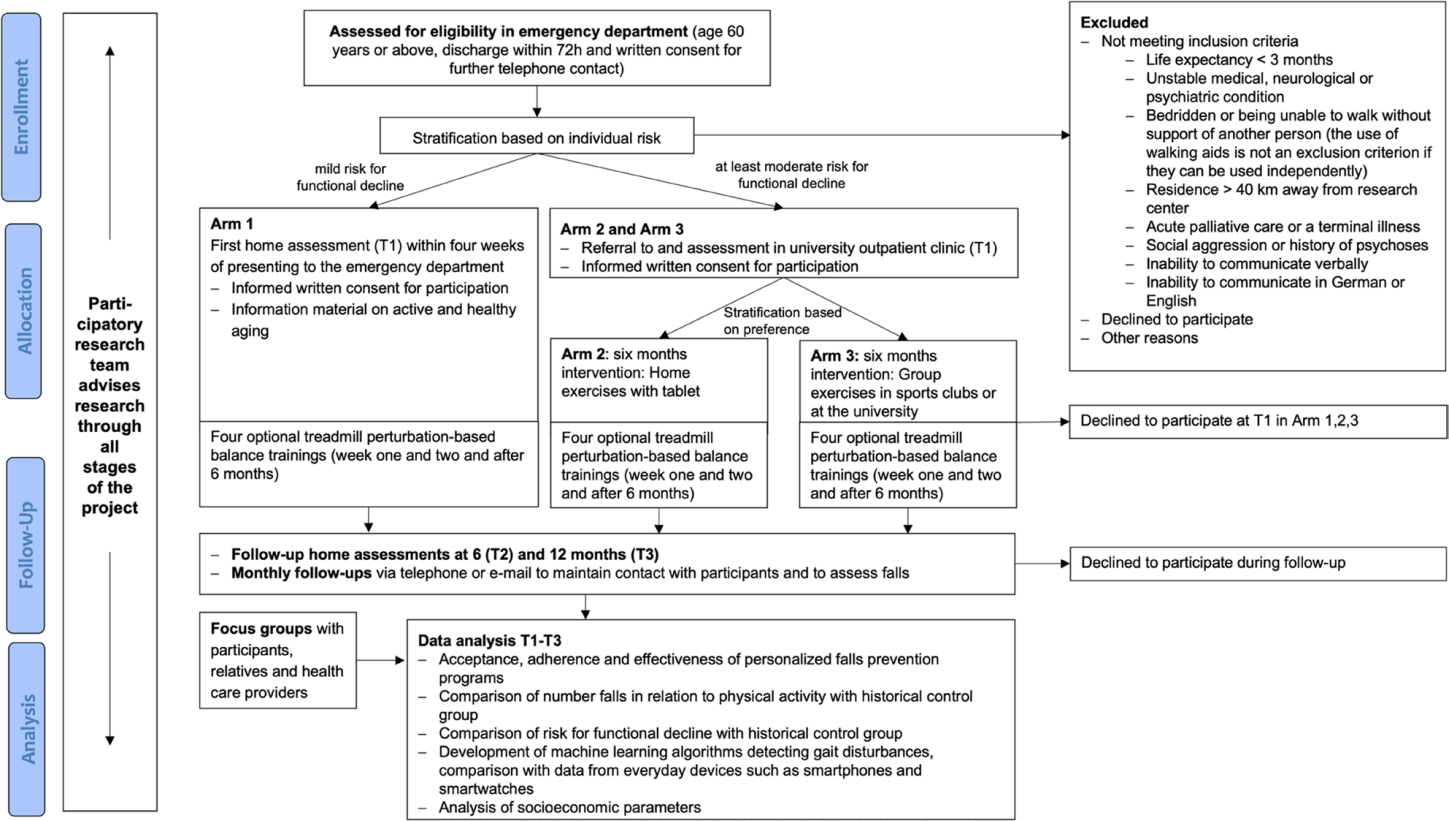
Flow diagram for the study design

Participants in the iSeFallED study will be followed for 12 months. The study will be conducted at the Carl von Ossietzky University in Oldenburg, Germany from October 2024 until September 2027, in accordance with the Declaration of Helsinki and was approved by the Medical Ethics Committee of the University of Oldenburg (number 2024–192). All participants will be asked to provide written informed consent to participate in the study. The consent forms are designed to be signed by either the participant or if needed by the participant and his / her legal representative. Ethical guidelines for research conducted with adults that lack the capacity to give consent will be followed and include the principle of group, the subsidiarity principle and the minimal risk standard [23]. Further, study information has been adapted to facilitate the participant’s understanding of the study, which will be provided in addition to the regular consent form, if necessary. Figure 1 shows the study design in a flow diagram.

### Implementation process

The iSeFallED study utilizes the RE-AIM framework (Reach, Efficacy, Adoption, Implementation, Maintenance), which was designed to evaluate interventions to improve general health, to guide and assess the implementation process [24]. The RE-AIM framework has been previously analyzed in a systematic review on physical activity-based falls prevention [25], concluding that all five key domains should be reported. In the iSeFallED study, these domains will be addressed as follows: *(1) Reach* – The study will assess the number and characteristics of patients identified as high risk in the ED and the proportion referred to the geriatric university outpatient clinic. Representativeness will be evaluated by comparing participant demographics with those of the broader ED population experiencing falls, the historical control group, and total admissions at the recruiting centers. *(2) Efficacy* – The efficacy of the intervention will be measured by evaluating functional outcomes, maintenance of daily activities, and secondary outcomes such as quality of life and fall recurrence. Additionally, unintended positive or negative effects will be documented. The outcomes will be compared with the historical study population of the SeFallED study, which underwent similar measurements. *(3) Adoption* – The study will analyze the extent to which healthcare professionals integrate the risk assessment tool and referral pathway into their workflow. Adoption at the institutional level will also be assessed, identifying facilitators and barriers to uptake. *(4) Implementation* – Regarding this dimension, adherence to the intervention will be evaluated at both provider and patient levels. It will assess the consistency of administering risk assessments and referrals in the ED, as well as patient participation in geriatric university outpatient clinic interventions, including adherence to prescribed exercise programs. Time and resource constraints affecting implementation will also be considered. *(5) Maintenance* – The study will examine the sustainability of integrating falls risk assessments and referrals into routine ED practice, as well as the long-term impact on patients, including continued engagement in falls prevention activities six months post-intervention. By systematically evaluating these dimensions, the iSeFallED study aims to provide a comprehensive understanding of the feasibility, effectiveness, and sustainability of embedding secondary falls prevention into ED care. In addition, the study will draw on key principles and guidance from the framework for developing and evaluating complex interventions to ensure a theoretically grounded and context-sensitive approach. This will support the translation of findings into practical strategies for broader implementation across diverse emergency care settings [26].

### Participants

Similar to the historic control group [22], participants will be recruited if they meet the following inclusion criteria: (1) age 60 years or above, (2) presented to the ED of the Klinikum Oldenburg or the Evangelisches Krankenhaus after a fall and discharged within 72 h, (3) provided written informed consent.

Exclusion criteria are as follows: (1) life expectancy of less than 3 months, (2) unstable medical, neurological or psychiatric condition, (3) bedridden or being unable to walk without support of another person, (4) residence more than 40 km away from the research center, (5) acute psychosis or social aggression, (6) inability to communicate verbally in German or English. By applying less restrictive inclusion and exclusion criteria, this study aims to better reflect real-world clinical care and improve the generalizability of findings compared to previous clinical research [27]. Importantly, efforts will be undertaken to also include people with cognitive impairment, which are excluded in most trials [11].

For participating in the treadmill perturbation-based balance training, additional exclusion criteria apply, arising from both personal and technical factors: (1) dependence on walking aids or inability to walk 400 m, (2) lower limb amputation or blindness, (3) recent osteosynthesis or joint replacement of the lower extremities performed within the past six weeks are exclusionary, as the uncontrolled movements inherent to treadmill perturbation-based balance training may increase the risk of luxation or dislocation, (4) significantly reduced bone density, (5) body weight exceeding 135 kg, which is based on the safety specifications of the treadmill manufacturer (Gait Real-time Analysis Interactive Lab system (GRAIL; Motek Medical B.V., CL Houten, The Netherlands)).

### Recruitment process

The recruitment process will take place in the EDs of Evangelisches Krankenhaus and Klinikum Oldenburg and builds on the recruitment strategy developed in the previous observational study [12]. Patients meeting the inclusion criteria will be screened by a member of the study team from Monday to Friday during regular working hours. Flyers containing study details and contact information will be distributed to patients outside the study teams’ working hours. Risk assessment and stratification will be conducted using routine ED data, analyzed through an algorithm developed based on findings from the preceding SeFallED study. This algorithm integrates individual characteristics, including age, use of walking aids, level of care, preexisting medical conditions, and educational level, with fall-related factors such as concerns about falling and history of previous falls. In addition, measures of subjective well-being and cognitive function are incorporated to classify participants into mild or at least moderate risk (manuscript in preparation). The assessment aims to categorize individuals according to their probability of experiencing functional decline following a fall.

Individuals at mild risk for functional decline (Arm 1) will be addressed by the study team in the ED to obtain written consent for further contact. Individuals consenting to further contact, will be contacted via telephone within a week of their ED visit. Those who consent to participate in the study will be scheduled for a home visit within four weeks. During this visit, the study team will explain the study details, allow sufficient time for participants to consider their involvement, and collect written informed consent. A comprehensive geriatric assessment will then be conducted in adherence to the protocol used in the SeFallED study [22].

For individuals identified as having moderate to high risk for functional decline (Arm 2 and 3), a referral to the geriatric university outpatient clinic located at Klinikum Oldenburg will be provided upon discharge from the ED, along with a recommendation to schedule a follow-up appointment within one week. If an appointment cannot be arranged at the time of discharge, individuals will be asked to consent to a follow-up contact by the study team via telephone. At the geriatric university outpatient clinic, participants will undergo a comprehensive geriatric assessment that includes an evaluation of their living situation, resources, and capabilities for participating in a secondary prevention program. Furthermore, a consultation with a geriatric specialist will be conducted, who will initiate further diagnostic procedures as needed. If patients agree to join the study, the study team will provide detailed information about the study procedures and obtain written consent. Participants may either sign the consent form on-site in the geriatric university outpatient clinic or return it later using a prepaid envelope.

### Intervention

The intervention is tailored based on risk stratification and personal preferences. Arm 1 includes participants at mild risk of functional decline. They receive evidence-based educational materials on falls prevention and healthy aging, aligned with the global guidelines for falls prevention [5]. These materials cover physical activity recommendation (e.g., 150 min of moderate activity or 75 min of vigorous activity per week, as well as strength and balance exercises), practical exercises for daily routines, and information on local sports programs in collaboration with local sports clubs. It also includes recommendations for avoiding falls in general life (e.g. adequate lightning at night, appropriate footwear, and reducing home hazards and basic information on medication use and polypharmacy risks), encouraging participants to discuss concerns with their general practitioner.

Participants with at least a moderate risk of functional decline are assigned to Arm 2 or Arm 3, based on personal preferences, assessed during their visit to the geriatric university outpatient clinic. Additionally, the consultation with a geriatric specialist during that visit provides an opportunity to address individual health concerns, including a critical medication review, which constitutes an integral component of the intervention in Arms 2 and 3.

In Arm 2, participants receive a home-based exercise program, delivered via tablet and designed by the research team. This progressive program focuses on strength, balance, and gait adaptation. Exercise begins with an in-person visit from the study team to ensure correct execution. Follow-up visits are conducted in weeks three and six for those requiring additional support, consistent with previous research approaches [28]. Participants will receive a total of nine different exercise videos developed by the study team, each offering varying levels of difficulty. During the first week of the intervention, participants will begin with the first video, followed by the second video in week two, continuing sequentially. After completing all nine videos over nine weeks, participants will restart with the first video. Each video lasts between 40 and 60 min, and participants will be encouraged to exercise at a moderate intensity, monitored using Borg’s Rating of Perceived Exertion (RPE) scale (between 13 and 16) [29].

Arm 3 offers supervised group-based exercises, either through local sports clubs or structured sessions at the University of Oldenburg, led by the study team. Sessions incorporate strength, balance, and gait adaption exercises. Participants in sports clubs receive a six-month membership reimbursement, while those attending university-based sessions are eligible for taxi fare reimbursement if mobility constraints prevent independent travel. To ensure training specificity, all participating sports clubs receive training from the study team as part of a workshop led at the university.

All participants, regardless of the study arm they participate in, are encouraged to exercise three times per week following World Health Organization recommendations [30]. Exercise intensity is monitored using Borg’s RPE. In Arms 2 and 3, exercise modifications are provided as needed. Participants receive exercise diaries to complete weekly and submit monthly via prepaid envelopes. Additionally, monthly phone calls serve as reminders and motivation to maintain physical activity. Attendance and participation in group training sessions are also documented by the trainer.

As an optional component, and within three months of study inclusion, all participants can undertake treadmill perturbation-based balance training consisting of four sessions lasting 30 to 45 min each, conducted on the Gait Real-time Analysis Interactive Lab system (GRAIL; Motek Medical B.V., CL Houten, The Netherlands). Treadmill belt speed is individually determined during a six-minute familiarization phase, following procedures similar to those used in previous research [22]. Familiarization begins at 50% of the participant’s preferred overground walking speed – assessed via a 3-meter walk test – and is gradually increased until the preferred treadmill walking speed is reached (maximum 100%). The preferred treadmill walking speed is determined within the first five minutes of walking, while the sixth minute is used to consolidate adaptation to the preferred walking speed and to record unperturbed walking.

Each training session comprises four blocks, each with a maximum duration of 3 min and 30 s, incorporating a minimum of 16 unannounced perturbations in randomized order per block. Perturbation types include lateral sway (left and right), pitch-up and pitch-down tilts, as well as single and double belt accelerations and decelerations. Perturbations are evenly distributed across the four perturbation types. A maximum of 15 difficulty levels is available depending on the type of perturbation. A detailed overview of the difficulty levels is provided in Table 1. Perturbations are triggered at the moment of initial foot contact, manually selected by a trained instructor, and occur every 8 to 11 s. This interval reflects the average time required for older adults to regain a stable gait pattern following a perturbation [31, 32].

In the third session, a dual task condition is introduced in two of the four training blocks. In the first dual-task block, participants are asked to verbally list items from specific categories while walking and reacting to perturbations – starting with fruits, followed by animals, and then words beginning with designated letters (S, B, L). In the second dual-task block, participants perform serial subtraction tasks: initially counting backward from 200 in steps of 7, and then from 400 in steps of 3.

This individualized, progressive treadmill perturbation-based balance training is tailored based on participant feedback, as well as perceived difficulty and anxiety using a scale from one (“easy” or “not at all”) to five (“too hard” or “extremely”) developed by Okubo and colleagues [33], with the aim of enhancing gait stability and evaluating its feasibility and acceptability in a diverse clinical population.

During each training session, the difficulty level, numbers of perturbations and any use of or falls into the safety harness will be documented, along with any adverse events (AEs) or serious adverse events (SAEs).

**Table 1:** The parameters for each difficulty level for the treadmill perturbation-based balance training. The speed increase and decrease refer to the percentage by which the speed of one or both sides of the treadmill belt is increased or decreased relative to the treadmill walking speed. The duration of acceleration/deceleration refers to the total time of the perturbation, including the time it takes to reach the maximum speed increase or decrease and the time during which this maximum speed is maintained. The duration of Tilt refers to the time taken to achieve the corresponding platform Tilt. The duration of sway refers to the time taken to reach the maximum sway

Difficulty Level	Acceleration/ Deceleration (m/s2)		Speed increase (%)	Duration of Acceleration (s)	Speed decrease (%)	Duration of Deceleration (s)	Tilt forward/ backward (degrees)	Duration of Tilt (s)	Sway Displacement (cm)	Duration of Sway (s)
1	3.5	30		0.5	10	0.5	1	1	5	0.18
2	4	45		0.5	20	0.5	2.5	1	-	-
3	4.5	60		0.5	30	0.5	4	1	-	-
4	5	75		0.5	40	0.5	5.5	1	-	-
5	5.5	90		0.5	50	0.5	7	1	-	-
6	6/ 6*	105/ 105*		0.5/ 0.5*	60/ 60*	0.5/ 0.5*	8.5	1	-	-
7	7/ 7*	120/ 120*		0.5/ 0.5*	70/ 70*	0.5/ 0.5*	10	1	-	-
8	8/ 8*	135/ 135*		0.5/ 0.5*	80/ 80*	0.5/ 0.5*	-	-	-	-
9	9/ 9*	150/ 150*		0.5/ 0.5*	90/ 90*	0.5/ 0.5*	-	-	-	-
10	10/ 10*	165/ 165*		0.5/ 0.5*	100/ 100*	0.5/ 0.5*	-	-	-	-
11	11/ 10*	180/ 180*		0.5/ 0.5*	100*	0.75*	-	-	-	-
12	12/ 10*	195/ 195*		0.5/ 0.5*	100*	1*	-	-	-	-
13	13/ 10*	210/ 210*		0.5/ 0.5*	-	-	-	-	-	-
14	14/ 10*	225/ 225*		0.5/ 0.5*	-	-	-	-	-	-
15	15/ 10*	240/ 240*		0.5/ 0.5*	-	-	-	-	-	-

*The parameters refer to all acceleration/deceleration perturbations performed simultaneously on both belts

### Data assessment

Data will be collected at the ED, in participants’ homes, in the geriatric university outpatient clinic and at the gait laboratory. Furthermore, Inertial Measurement Units (IMUs) to measure physical activity as well as gait parameters will be worn by the participants during daily life over seven days at each time point (T1 – T3). After the initial assessment, the researchers maintain contact with the participants through monthly phone calls, as part of the prospective falls recording in addition to fall calendars (Fig. 1). First data assessment will be within four weeks of presenting to the ED. Whereas participants in Arm 1 will be visited at home for the first functional assessment (T1), participants in Arms 2 and 3 will be assessed in the geriatric university outpatient clinic and additionally via telephone interview. Data collection after the intervention (T2) and twelve months after presenting to the ED (T3) will be scheduled at participants’ homes.

### Outcome measures

As part of a comprehensive geriatric assessment, participants’ characteristics, i.e. demographic data, socioeconomic status, medical history, medication use, sensory and mobility aid use, and lifestyle factors will be recorded. A detailed fall history, including circumstances and consequences of the most recent falls, will be collected. Validated instruments will be used to evaluate fall risk, functional status, cognitive performance, depressive symptoms, physical activity, and health-related quality of life. Exercise participation and adherence will be monitored and falls will be tracked throughout the follow-up period. Additionally, sensor-based data regarding physical activity will be collected. An overview of all assessment tools and time points is provided in Table 2.

**Table 2:** Assessment battery during the emergency department Visit, home visits, geriatric university outpatient Clinic, visits and training sessions

Assessment tool	Outcomes	Timepoint
1. Risk stratification	Age, gender, country of birth, highest level of education, level of care, living situation, medical care, capacity to get up after falling	Emergency department
2. Specific fall history	Time of fall, location of fall, activity before falling, direction of fall, injuries, loss of consciousness, capacity to get up after fall	T1/ clinic^+^
3. Socioeconomic status	Income, education [34], occupation [34], satisfaction with living situation [35]	T1/ clinic^+^
4. Participants’ characteristics (including personal and medical history)	Usage of hearing, seeing and walking aids, living situation, smoking status, alcohol consumption, medication, joint replacements.	T1/ clinic^+^, T2, T3
5. Fall risk assessment tool (FRAT-up) [36]	Total risk score (ranging from 0–1)	T1, T2, T3
6. (instrumental) activities of daily living a. Lawton’s and Brody’s Index [37] b. Barthel Index [38] c. Jonkmann Index [39]	Total score (ranging from 0–8) Total score (ranging from 0–100) Total score (ranging from 0–18)	T1/ clinic^+^, T2, T3
7. Functional performance: a. Hand grip strength test [40] b. Single leg stance test [41] ** c. Short Physical Performance Battery Test (SPPB) [42]**	Grip strength measured in kg Duration in seconds Total score (ranging from 0–12)	T1/ clinic^+^, T2, T3
8. German short falls efficacy scale (short FES-I) [43, 44]	Total score (ranging from 7–28)	T1/ clinic^+^, T2, T3
9. Montreal Cognitive Assessment (MoCA) [45] a. MoCA memory index score (MoCA-MIS) [45, 46]	Total score (ranging from 0–30) Total score (ranging from 0–15)	T1/ clinic^+^,T2, T3
10. Trail Making Test A and B (TMT A + B) [47]	Duration until completion in seconds & number of mistakes	T1/ clinic^+^,T2, T3
11. Longitudinal Urban Cohort Ageing Study (LUCAS - FI) Functional Ability Index (LUCAS - FI) [48]	Functional ability classified in: Robust, postRobust, preFrail, Frail	T1/telephone interview^++^, T2, T3
12. Physical activity: a. German-Physical-Activity-Questionnaire 50+ (PAQ-50+) [49] b. Physical Activity Scale for the Elderly (PASE) [50] c. Activity monitor (activPAL^©^) worn for 7 days	Energy expenditure per week Total score (ranging from 0–793) Number of steps, total active / inactive time	T1, T2, T3
13. German Life Space Questionnaire (LSA-D) [51, 52]	Total score (ranging from 0–120)	T1/telephone interview^++^, T2, T3
14. Depressive Symptoms a. Depression in Old Age Scale (DIA-S) [53] b. Cornell Depression Scale [54]*	Total score (ranging from 0–10) Total score (ranging from 0–38)	T1/ clinic^+^, T2, T3
15. Health-related quality of life EQ-5D – 3 L + EQ-5D Visual Analogue Scale [55, 56]	Total score (ranging from 0–15)	T1/ clinic^+^, T2, T3
16. Loss of Balance documentation	Total number of balance losses during daily life, self-reported for seven days	T1, T2, T3
17. Fall Calendar	Total number of falls during the follow up period	Monthly phone calls
18. Training characteristics a. Borg scale [57]	Frequency of participation in training, duration and contents of training Total score (ranging from 6–20)	Every training session
19. Perturbation training characteristics*** a. Perceived difficulty and anxiety [33]	Gait speed, duration of training session, type of perturbations, number of perturbations, dynamic reactive balance Total score (ranging from 1–5)	Every gait lab training session

* The Cornell Depression Scale will be used instead of the DIA-S in case of severe cognitive impairment (MoCA < 18) or an existing diagnosis of dementia

** During the functional assessments, participants will be equipped with three wirelessly synchronized IMUs(Opal V1, Mobility Lab™, APDM, Inc., Portland, OR, USA), which will objectively assess postural sway and gait characteristics [58, 59]

*** During the gait lab visits, participants will wear the IMUs, alongside an activPAL monitor (activPAL4™, PAL Technologies Ltd., Glasgow, UK), as well as a wrist-worn IMU (Axivity AX6, Axivity Ltd., Newcastle upon Tyne, UK) and a smartphone (Galaxy A52, Samsung Electronics Co., Suwon, South Korea) positioned on the body

Clinic^+^: arm 2 and 3, baseline parameters are assessed at the geriatric university outpatient clinic

Telephone interview^++^, parameters are collected via telephone interview

### Focus groups

To explore preferences, needs, facilitators, and barriers associated with the implementation of secondary falls prevention in clinical settings, as well as to evaluate participants’ acceptance of the implementation process from a patient-centered perspective, focus group interviews will be conducted as part of the study [60]. The study aims to recruit 72 individuals, including participants, relatives/caregivers and stakeholders, who will be assigned to twelve focus groups (six with participants, three with relatives, three with stakeholders), which is in line with previous approaches [22].

Informed consent for potential participation in a focus group will be obtained during the initial home visit or at the university outpatient clinic. The consent form will ask participants to specify whether they agree to be contacted again for this purpose. Participants who consented to be contacted for participation in a focus group will then be chosen purposefully, depending on criteria like their study arm, to create matching group constellations to engage discussions.

During the initial home visit or visit in the geriatric university outpatient clinic, participants will be offered a flyer inviting relatives to participate in a focus group discussion. Relatives may voluntarily reach out and, upon agreeing to take part, will receive an informed consent form to confirm their participation. Similarly, members of relevant organizations will also receive flyers inviting them to participate in focus groups. If they make contact, the necessary informed consent documentation is provided.

Focus groups will last 90 min and participants of the focus group will receive financial compensation for the spent time. Audio data from the focus group interviews will be transcribed with two multidisciplinary independent researchers analyzing the data. Content analysis according to Kuckartz [61] will be applied with categories such as needs, preferences, barriers, and facilitators deductively derived from the Theoretical Domains Framework [62] and the Consolidated Framework for Implementation Research [63]. Furthermore, categories will be derived inductively from the material recorded [64].

### Sensor data acquisition and machine learning

In the historical control study SeFallED, machine learning algorithms were developed to automatically detect gait disturbances induced by standardized perturbations in the gait laboratory [65]. Subsequent work demonstrated that everyday wearable devices positioned at typical body locations, such as smartphones and hearing aids, can also support automatic detection of such disturbances [66, 67]. In the iSeFallED study, those algorithms, in particular deep convolutional long short-term memory network (DeepConvLSTM), will be further refined using an extended perturbation protocol that incorporates varying perturbation intensities and a larger number of repetitions (Table 1); these modifications are expected to enhance algorithm robustness and generalizability. Furthermore, different approaches will be explored: spectrogram-based analyses will be applied to identify characteristic temporal-frequency signatures, and anomaly-detection methods will be explored to prescreen events of interest.

The following sensor systems will be worn during the treadmill perturbation-based balance training sessions performed in the laboratory. Treadmill-based sensor instrumentation will include: a thigh-worn accelerometer (activPAL4, PAL Technologies Ltd., Glasgow, UK); an inertial measurement unit (IMU) comprising accelerometer, gyroscope and magnetometer (Opal V1, Mobility Lab, APDM Inc., Portland, OR, USA) worn at six body positions (feet, wrists, lumbar and sternum); a wrist-worn device resembling a smartwatch position with accelerometer and gyroscope (Axivity AX6, Axivity Ltd., Newcastle upon Tyne, UK); a hearable device with integrated accelerometer (Cosinuss c-med° alpha, Cosinuss GmbH, Munich, Germany); and a smartphone (Galaxy A52, Samsung Electronics Co., Suwon, South Korea) to capture accelerometer data similar to a phone worn in the right pocket of the trouser. The Opal IMU and the hearable device will be used exclusively in the laboratory and will not be deployed in at-home monitoring.

The at-home component of the study is exploratory and specifically aims to assess whether perturbations detected in the laboratory can also be transferred in everyday life using everyday wearable devices and following up on previous work by Feld and colleagues [66, 67]. For this purpose, a convenience sample of approximately 100 participants - consistent with prior work by Hellmers and colleagues [65] - will be monitored continuously for seven consecutive days. In the at-home monitoring phase, participants will wear the wrist-worn activity monitor (Axivity AX6, Axivity Ltd., Newcastle upon Tyne, UK), a thigh-worn activity monitor (activPAL4, PAL Technologies Ltd., Glasgow, UK), and carry a smartphone (Galaxy A52, Samsung Electronics Co., Suwon, South Korea). These IMUs and the smartphone will continuously record movement data over the full seven-day period. To aid in identifying episodes of balance loss, participants will keep a daily diary documenting any occurrences of loss of balance (for example, tripping, slipping, or stumbling). The combined laboratory and at-home datasets will enable (1) within-laboratory algorithm refinement across multiple body positions and sensor types, and (2) an exploratory assessment of the detectability of gait perturbations in real-world settings.

### Safety

All serious adverse events (SAEs) and adverse events (AEs) will be documented using study-specific adverse event reporting forms. Overexertion during training will be mitigated by offering modifications for strenuous exercises and implementing Borg’s RPE scale, which has been shown to be feasible for older adults with and without cognitive impairment [29, 68]. The individualized nature of the training protocol will accommodate the heterogeneous needs of participants, ensuring a tailored approach.

In particular, treadmill perturbation-based balance training will be guided by a scale that considers both perceived difficulty as well as anxiety levels, as previously recommended for applying treadmill perturbation-based balance training [33]. Additionally, participants will be regularly asked whether they feel fit to continue, and they may request a break at any time if needed. If subjective or objective indicators of overexertion are observed, the training session will be paused. All adverse events will be promptly reported to the principal investigator.

### Statistical analysis and sample size calculations

Descriptive analyses will summarize participant characteristics, including age, sex, place of residence, level of education, and intervention choice. Differences between groups in these characteristics, as well as adherence to the assigned or selected interventions and the proportion of participants who attend the health sport activities, will be explored to assess feasibility and representativeness.

Feasibility of the training interventions will be assessed by documenting AEs and SAEs, as well as dropout rates, while acceptability will be evaluated through focus groups. The effectiveness of the tailored interventions in the iSeFallED study will be analyzed using a Wilcoxon-Mann-Whitney test, comparing endpoints between the historical control group (SeFallED) and the new study population. The primary outcome will be the change in Activities of Daily Living (ADL) scores between T2 (6 months) and T3 (12 months) using ADL scores from Jonkman and colleagues [39].

An independent statistician performed the sample size calculation to assess effectiveness. This calculation was based on data from 257 participants in the previous SeFallED project, who were followed over a 12-month period. For the iSeFallED study, it was assumed that the tailored interventions would result in a clinically meaningful improvement of at least 1 point in ADL score in individuals with at least a moderate risk of functional decline [39]. No difference is expected for individuals in Arm 1 (mild risk) compared to the historical control group. Data from the previous study indicated that approximately one-third of participants exhibited a low risk of functional decline (Arm 1), while two-thirds had at least a moderate risk (Arms 2 and 3). Consequently, an expected difference (Δ) of 0.67 points in the ADL score was determined for the new study (iSeFallED).

To detect a meaningful difference in ADL scores between T2 and T3 with a power of 90% at a significance level of α = 0.05, a total of 246 participants is required. Accounting for an estimated dropout rate of approximately 20% (based on a 17% dropout rate in the previous study), the necessary sample size increases to 308 participants. To further strengthen the study’s robustness and allow for secondary, exploratory analyses, a total sample size of 350 participants is planned.

Secondary endpoints include fall rate, time to first fall, and the number of falls in relation to physical activity compared to the historical control group. This analysis aims to evaluate the effectiveness of the interventions in preventing further falls. Furthermore, socioeconomic factors will be analyzed at both the regional and individual levels. The effects of the secondary falls prevention program on further secondary endpoints such as health-related quality of life, life-space mobility, physical activity, and cognitive function will also be explored in comparison with the historical control group.

### Data management

Data will be managed using unique and pseudonymized study codes. These will be used to code and file all electronic information that will be stored on secured university systems. Paper and pencil tests will be stored in a cabinet with a lock. Data will be integrated into a custom-made Research Electronic Data Capture (REDCap) database hosted at the Carl von Ossietzky University Oldenburg. Data will be collected via REDCap Mobile App using a tablet owned by the university. REDCap is a secure, web-based software platform, designed to support data capture for research studies [69, 70].

### Patient and public involvement

A participatory research team (PRT), which was approved by the Medical Ethics Committee of the University of Oldenburg (number 2024–160), will be involved as consultants. The PRT consists of older adults, who have themselves experienced a fall before, and individuals from the health sector, such as exercise group instructors or counseling centers for individuals with cognitive impairment. Four members of the PRT already took part in the previous study and provided feedback to study planning. Approximately 26 meetings between researchers and the PRT will take place throughout the course of the iSeFallED study. The PRT will provide feedback for example to the comprehensibility of consent forms, the burden of the assessment batteries, strategies of participant recruitment and the guidelines for the focus group interviews. All meetings between the PRT and the researchers will be documented with focus on the remarks of the PRT. The PRT’s feedback, and whether and to what extent it will lead to changes in study procedures, will be documented. Furthermore, semi-structured telephone interviews will be performed with all members of the PRT to evaluate their integration and participation in the research process. An independent researcher, not participating in meetings with the PRT, will conduct the interviews. Audio data from interviews will be transcribed with two multidisciplinary independent researchers analyzing the data. Content analysis according to Kuckartz [61] will be applied with deductively derived categories from previous studies about PRTs and inductively derived categories from the recorded material [64]. Members of the PRT will receive an expense allowance of 25€ per hour according to documented working hours, which include meetings and working tasks between meetings.

## Discussion

Falls represent a major public health challenge, particularly in the context of an ageing population. Although effective strategies for both primary and secondary falls prevention exist, their implementation remains difficult. This is especially evident in clinical settings, where barriers such as limited financial and personnel resources, as well as the heterogeneity of patient populations, hinder the delivery of evidence-based interventions. The iSeFallED study aims to address these challenges by evaluating the implementation of a tailored secondary falls prevention program for older individuals presenting to the ED following a fall.

The present study employs a risk stratification scheme specifically adapted for the ED context, using data from a previous observational study that followed a comparable study population using similar inclusion and exclusion criteria [22]. Implementing risk stratification is essential for delivering tailored interventions – both to optimize healthcare resource allocation and to meet individual functional as well as personal abilities. The importance of fast fall risk identification has been emphasized by global guidelines for falls prevention, which introduced a risk stratification scheme for older adults [5]. While this may represent an important milestone in patient care, such schemes have been critically discussed as they often lack adaptability to local contexts and resources [71]. Moreover, their applicability to specific patient cohorts may be challenging, prompting several refinements of the original framework to improve predictive accuracy [72, 73]. In the original guideline-based scheme, all individuals presenting to emergency services after a fall are classified as having experienced a severe fall, thereby being deemed at high risk for future falls and functional decline. However, data from the observational study SeFallED suggests that this is not always the case – particularly for individuals discharged directly from the ED without further inpatient care [74]. For this subgroup, a distinct risk stratification approach may provide greater accuracy and contextual fit. Therefore, the iSeFallED study will assess whether ED-specific risk stratification derived from the SeFallED data is both feasible and accurate, and whether it is acceptable to participants.

To evaluate feasibility, efficacy, and implementation procedure, iSeFallED adopts a pragmatic mixed methods trial design. Quantitative measures – including activities of daily living, monthly fall incidence (via telephone follow-up), adverse events, and dropout rates – will assess feasibility and efficacy. Acceptance within the target population will be assessed qualitatively through focus groups similar with previous approaches [75–78]. Integrating multiple data sources is essential to advance understanding of secondary falls prevention strategies. To ensure alignment with priorities of the patients and healthcare providers, a PRT – including stakeholders, patient representatives, and health service providers – will support the study. Early involvement of these groups can enhance the relevance, design, and acceptability of interventions. In health services research, such participatory approaches are increasingly recognized for improving recruitment, fostering relevance, and enhancing research quality [79, 80].

A further strength of the iSeFallED study lies in the diversity of its intervention delivery. While the falls prevention interventions focus on strength and balance training – reflecting the strongest evidence identified in the global guidelines for falls prevention [5] – its delivery is tailored to individual needs and preferences, offering both home-based and group-based solutions either at the university center or in local sports clubs. For participants at mild risk, iSeFallED offers education and advise on falls prevention and physical activity, which is recommended by the global guidelines for falls prevention [5]. Furthermore, to include an innovative approach, the study offers treadmill perturbation-based balance training to all participants. This task-specific training of dynamic balance response has shown promising effectiveness in previous studies [6, 7], and its feasibility in an unselected, heterogenous clinical population will be evaluated.

Incorporating treadmill-based perturbations also enables further refinement of the previously developed machine learning algorithms [65, 67]. Automated detection of gait disturbances could improve early identification of individuals at risk for recurrent falls and enable more targeted intervention prescriptions. This approach aligns with a recent call to action from the European Geriatric Medicine Society Special Interest Group on Falls and Fractures, which emphasizes both the translation of evidence-based recommendations into real world settings and the advancement of technology-assisted systems [81]. By evaluating data from wearable devices such as smartphones, smartwatches, and hearables, the study will provide novel insights into detecting gait disturbances in daily life, using routinely worn monitors.

Successful recruitment and sustained adherence are key factors to the success of the study, with participant drop-out during the 12-month follow-up presenting a substantial risk. To address this, a well-established and previously evaluated recruitment process will be implemented, with research personnel present in the ED Monday through Friday to engage directly with potential participants [12]. In addition, nurses and physicians in the ED will receive training to distribute study information flyers to patients who meet the inclusion and exclusion criteria outside of working hours of the research team. A centralized point of contact for individuals at risk – via a geriatric university outpatient clinic – will be provided, in accordance with prior research recommendations [17]. By offering tailored interventions, the study aims to optimize the “burden to benefit ratio” for participants, which may improve recruitment and retention compared with the previous observational study (SeFallED). Secondary analyses will assess whether the different intervention deliveries yield comparable benefits. As standardization is more challenging in home-based programs and community sport club settings than in the laboratory setting of an intervention undertaken by the study staff itself, iSeFallED will provide important insights into the relative effectiveness of varied delivery approaches.

The iSeFallED study will employ the study participant cohort from the prior SeFallED study as a historical control group [82], thereby addressing the well-recognized recruitment challenges associated with vulnerable patient populations. This design allows the study to better reflect real-world clinical practice, as RCTs frequently exclude a substantial proportion of patients, and, thus, limit generalizability. By maintaining inclusion and exclusion criteria consistent with those used in the previous study, iSeFallED seeks to extend understanding of the clinical effectiveness of secondary falls prevention within routine healthcare contexts [27, 83]. While this approach increases the effective sample size and enhances external validity, several limitations must be acknowledged. Participants enrolled during the earlier study may have been subject to different global or local circumstances, which could influence outcome trajectories. To mitigate potential biases inherent to the use of historical controls [82, 84], the baseline characteristics between the two study populations will be systematically compared, and statistical models will be adjusted for any identified differences by the study biostatistician.

The findings of iSeFallED will inform the feasibility, acceptability, and effectiveness of a secondary prevention program for older individuals presenting to the ED after a fall but not requiring hospitalization. The study will also evaluate implementation success within the complex and resource-limited ED environment, offering opportunities to refine processes to improve outreach and inclusivity in healthcare services for older adults. Furthermore, it will refine automated gait disturbance detection using machine learning algorithms and assess its feasibility in real-life data. Through its mixed-methods design, iSeFallED has the potential to serve as a model for integrating secondary falls prevention interventions into clinical practice, ensuring accessibility and scalability for a broader population.

## Supplementary Information

Supplementary Material 1.

## Data Availability

Data sharing is not applicable as no datasets were generated or analyzed for this article.

## Abbreviations

ADL: Activities of Daily Living
AE: Adverse Event
DeepConvLSTM: Deep Convolutional Long Short-Term Memory Network
DIA-S: Depression in Old Age Scale
ED: Emergency Department
FES-I: Falls Efficacy Scale
FI: Functional Ability Index
GRAIL: Gait Real-time Analysis Interactive Lab
IMU: Inertial Measurement Unit
LSA-D: German Life Space Questionnaire
LUCAS: Longitudinal Urban Cohort Ageing Study
MoCA: Montreal Cognitive Assessment
MIS: Memory Index Score
PASE: Physical Activity Scale for the Elderly
PRT: Participatory Research Team
QoL: Quality of Life
RPE: Rating of Perceived Exertion
RE-AIM: Reach, Efficacy, Adoption, Implementation, Maintenance
REDCap: Research Electronic Data Capture
RCT: Randomized Controlled Trial
SeFallED: Sentinel Fall Presenting to the Emergency Department
SAE: Serious Adverse Event
SPPB: Short Physical Performance Battery
iSeFallED: Tailored Interventions to Prevent Functional Decline After a Sentinel Fall Presenting to the Emergency Department
TMT: Trail Making Test
UK: United Kingdom
USA: United States of America
WHO: World Health Organization
